# Minocycline modulates NFκB phosphorylation and enhances antimicrobial activity against *Staphylococcus aureus* in mesenchymal stromal/stem cells

**DOI:** 10.1186/s13287-017-0623-1

**Published:** 2017-07-21

**Authors:** Alberto Daniel Guerra, Warren E. Rose, Peiman Hematti, W. John Kao

**Affiliations:** 1 School of Pharmacy, Division of Pharmaceutical Sciences, Pharmacy Practice Division, University of Wisconsin-Madison, 777 Highland Avenue, 7123 Rennebohm Hall, Madison, WI 53705 USA; 2 School of Medicine and Public Health, Department of Medicine, Wisconsin Carbone Cancer Center, University of Wisconsin-Madison, 1685 Highland Avenue, Madison, WI 53705 USA; 3 College of Engineering, Department of Biomedical Engineering, University of Wisconsin-Madison, 1415 Engineering Drive, Madison, WI 53706 USA; 4 School of Medicine and Public Health, Department of Surgery, University of Wisconsin-Madison, 1685 Highland Avenue, Madison, WI 53705 USA; 5 Present Address: 10/F Knowles Building, Pokfulam, Hong Kong

**Keywords:** Mesenchymal stromal cells, Mesenchymal stem cells, Biomaterials, Minocycline, NFκB, Antimicrobial, Hydrogel, Animal model

## Abstract

**Background:**

Mesenchymal stromal/stem cells (MSCs) have demonstrated pro-healing properties due to their anti-inflammatory, angiogenic, and even antibacterial properties. We have shown previously that minocycline enhances the wound healing phenotype of MSCs, and MSCs encapsulated in poly(ethylene glycol) and gelatin-based hydrogels with minocycline have antibacterial properties against *Staphylococcus aureus* (SA). Here, we investigated the signaling pathway that minocycline modulates in MSCs which results in their enhanced wound healing phenotype and determined whether preconditioning MSCs with minocycline has an effect on antimicrobial activity. We further investigated the in-vivo antimicrobial efficacy of MSC and antibiotic-loaded hydrogels in inoculated full-thickness cutaneous wounds.

**Methods:**

Modulation of cell signaling pathways in MSCs with minocycline was analyzed via western blot, immunofluorescence, and ELISA. Antimicrobial efficacy of MSCs pretreated with minocycline was determined by direct and transwell coculture with SA. MSC viability after SA coculture was determined via a LIVE/DEAD® stain. Internalization of SA by MSCs pretreated with minocycline was determined via confocal imaging. All protein and cytokine analysis was done via ELISA. The in-vivo antimicrobial efficacy of MSC and antibiotic-loaded hydrogels was determined in Sprague–Dawley rats inoculated with SA. Two-way ANOVA for multiple comparisons was used with Bonferroni test assessment and an unpaired two-tailed Student’s *t* test was used to determine *p* values for all assays with multiple or two conditions, respectively.

**Results:**

Minocycline leads to the phosphorylation of transcriptional nuclear factor-κB (NFκB), but not c-Jun NH_2_-terminal kinase (JNK) or mitogen-activated protein kinase (ERK). Inhibition of NFκB activation prevented the minocycline-induced increase in VEGF secretion. Preconditioning of MSCs with minocycline led to a reduced production of the antimicrobial peptide LL-37, but enhanced antimicrobial activity against SA via an increased production of IL-6 and SA internalization. MSC and antibiotic-loaded hydrogels reduced SA bioburden in inoculated wounds over 3 days and accelerated reepithelialization.

**Conclusions:**

Minocycline modulates the NFκB pathway in MSCs that leads to an enhanced production of IL-6 and internalization of SA. This mechanism may have contributed to the in-vivo antibacterial efficacy of MSC and antibiotic-loaded hydrogels.

**Electronic supplementary material:**

The online version of this article (doi:10.1186/s13287-017-0623-1) contains supplementary material, which is available to authorized users.

## Background

Bone marrow-derived mesenchymal stromal/stem cells (MSCs) have demonstrated pro-healing properties in addition to their differentiation potential. Such pro-healing properties include an anti-inflammatory cytokine expression profile, decreased fibrotic granular tissue formation, enhanced keratinocyte proliferation, anti-myofibroblast differentiation, elevated secretion of growth factors and proliferation of microvascular endothelial cells with subsequent angiogenesis, and even antibacterial properties [1–10]. MSCs encapsulated in poly(ethylene glycol) and gelatin-based hydrogels for spatially and temporally controlled cellular presentation to wound sites have demonstrated the promotion of full-thickness cutaneous wound healing [11–14]. Extracellular matrix (ECM)-incorporated scaffolds for MSCs such as these hydrogels have been shown not only to enhance cellular delivery, but also to enhance the bioactivity of biomaterial scaffolds for MSCs through enhanced proliferation and migration [15]. Hydrogels loaded with minocycline alone or in combination with vancomycin and linezolid in addition to MSCs have been shown to prevent the bioburden of *Staphylococcus aureus* (SA) infection [16, 17]. Interestingly, minocycline has been shown to enhance the wound healing phenotype of MSCs in culture and in hydrogels that includes increased invasion capacity, proliferation, ECM attachment, adhesion molecule production, and growth factor production with subsequent angiogenesis [17]. However, the mechanism of MSC wound healing phenotype enhancement has not yet been investigated in detail.

MSC behavior and ultimate expression of wound healing properties is governed by the activation of signaling pathway proteins such as mitogen-activated protein kinase (MAPK/ERK), transcriptional nuclear factor-κB (NFκB), and c-Jun NH_2_-terminal kinase (JNK/SAPK) [18–20]. Previous studies have demonstrated that the NFκB and MAPK pathways can be activated due to the stimulation of injury or inflammatory factors, such as TNF-α, that leads to some of the pro-healing phenotype enhancements we have observed in MSCs after treatment with minocycline. These observed effects include an increase in growth factor secretion, adhesion molecule expression, and cytokine production [17, 18, 21–23]. Additionally, MSCs have demonstrated an increase in proliferation, migration, and growth factor secretion through the MAPK pathway activation from the antibacterial peptide LL-37 produced by MSCs that has also been shown to contribute to MSC antibacterial potential against *Escherichia coli* and SA [9, 10, 24, 25]. In this study, we investigate the signaling pathway that is modulated by minocycline in MSCs. We then determine whether minocycline has an effect on the MSC production of LL-37 that could have contributed to the MSC enhancement observed previously. We demonstrate that minocycline modulates the phosphorylation of the NFκB pathway in MSCs but inhibits LL-37 production, which led us to investigate the antimicrobial effects of MSCs pretreated with minocycline. We show that MSCs pretreated with minocycline have a significantly enhanced antibacterial capacity against SA due to an increase in IL-6 production and enhancement of SA internalization. Lastly, we demonstrate the in-vivo antibacterial efficacy of minocycline, vancomycin, linezolid, and MSC-loaded hydrogels in SA-inoculated full-thickness cutaneous wounds.

## Methods

### MSC isolation, characterization, and culture

MSCs were isolated from discarded filters of bone marrow harvests of healthy adult human donors based on a protocol approved by the University of Wisconsin Hospital and Clinics Regulatory Committee per our published protocols [12, 26]. Isolated MSCs at passage 4 were characterized for positive and negative markers via flow cytometry, and for multidifferentiation potential as described previously [12, 26, 27]. MSCs were cultured in 75-cm^2^ tissue culture flasks (TPP, St. Louis, MO, USA) with Dulbecco’s Modified Eagle Medium (DMEM; Cellgro Mediatech, Inc., Corning, NY, USA), 10% fetal bovine serum (FBS), 2 mM l-glutamine, and 2 mM nonessential amino acids (NEAA) with medium changes every 3–5 days. Only MSC passages 4–8 were used in this study.

### Minocycline treatment, protein extraction, and western blot

MSCs were cultured in six-well tissue culture plates (CellTreat, Shirley, MA, USA) at 300,000 wells/well in triplicate with 2 ml of MSC culture medium at 0, 50, or 100 μg/ml minocycline (Research Products International, Mt. Prospect, IL, USA) for 48 hours. MSCs were then harvested and protein was extracted using a NE-PER® Nuclear and Cytoplasmic Extraction Kit with Halt™ protease and phosphatase inhibitor cocktail (Thermo Fischer Scientific). Cytoplasmic protein extract concentrations were determined using a *DC*™ Protein Assay (BioRad, Hercules, CA, USA) and the presence of specific proteins in the extracts was determined using a western blot analysis of 10 μg of protein as described previously [28]. Primary antibodies used were from Cell Signaling Technologies (Danvers, MA, USA): monoclonal rabbit α-phospho-NF-kappaB (catalog no. 3033), monoclonal rabbit α-NF-kappaB (catalog no. 8242), monoclonal rabbit α-phospho-JNK2 (catalog no. 9251), monoclonal α-JNK2 (catalog no. 9258), monoclonal rabbit α-phospho-MAPK (ERK 1/2) (catalog no. 4370), monoclonal rabbit α-MAPK (ERK 1/2) (catalog no. 4695), and monoclonal α-beta-actin (catalog no. 4970) all at 1:1000. The secondary antibody used was alkaline phosphatase-conjugated goat α-rabbit IgG (1:30,000; GE Healthcare, Little Chalfont, UK). Membranes were developed for imaging with ECF substrate (GE Healthcare) and imaged on a Storm 840 Scanner (Amersham Bioscience, Amersham, UK) with ImageQuant TL software version 7.0 (GE Healthcare).

### Immunofluorescence

MSCs were cultured overnight in six-well tissue culture plates (CellTreat) with glass coverslips added to the wells at 175,000 cells/well with 2 ml of MSC culture medium overnight. MSCs were then cultured with 0, 50, or 100 μg/ml minocycline for 24 hours. After treatment, media were removed and MSCs were washed three times with PBS. MSCs were then fixed for 10 minutes with 4% paraformaldehyde and washed once with PBS. The slides were blocked for 40 minutes using 4% BSA in TBS and then treated with the same primary rabbit α-phospho-NF-kappaB antibody stated earlier at 1:200 in 4% BSA in TBS at 4 °C overnight. The MSCs were washed four times for 10 minutes with TBS and then treated with goat anti-rabbit IgG Alexa Fluor 488 secondary antibody (ThermoFisher Scientific) at 1:200 in 4% BSA for 30 minutes at room temperature. MSCs were then washed twice for 10 minutes with TBS and stained with DAPI for 2 minutes, covered with a coverslip coated with VECTASHIELD® mounting medium, and imaged on an Olympus Fluoview FV1000.

### NFκB phosphorylation and protein production analysis

Phosphorylation of NFκB residues was determined using a FACE™ NFκB p65 profiler (Active Motif, Carlsbad, CA, USA). Briefly, MSCs were plated at 50,000 cells/well overnight and then treated with or without minocycline for 24 hours and the protocol was continued using the manufacturer’s instructions. MSC proliferation was determined using the crystal violet stain included in the kit. VEGF, IL-6, TNF-α, and MCP-1 analysis was carried out using a DuoSet® ELISA kit system (R&D Systems, Minneapolis, MN, USA). LL-37 analysis was performed using an ELISA kit (Hycult Biotech, Uden, the Netherlands). Complement (C5, C5a, C3, C3b) analysis was performed using respective ELISA kits (Abcam, Cambridge, MA, USA). For VEGF analysis, MSCs were cultured in 24-well tissue culture plates (CellTreat) at 100,000 cells/well in triplicate with 1 ml of MSC culture medium at 0, 50, and 100 μg/ml minocycline with or without 10 μM of the NFκB inhibitor PDTC (BioVision, Milpitas, CA, USA) for 24 hours prior to analysis. For LL-37 production, a treatment condition was used where MSCs were stimulated with IFNγ (100 ng/ml; Sigma Aldrich) for 24 hours [10].

### MSC cocultures with *Staphylococcus aureus*

MSCs were cultured overnight in 48-well tissue culture plates (CellTreat) at 50,000 cells/well with 0.5 ml of MSC culture medium. After overnight culture, media were removed and replaced with either fresh MSC media alone, fresh MSC media at 100 μg/ml minocycline, or fresh MSC media at 80 μg/ml vancomycin and cells were cultured for 24 hours. SA (strain #29213; ATCC, Manassas, VA, USA) culture vials stored at −80 °C were thawed, plated on trypticase soy agar (TSA; Becton Dickinson, Sparks, MD, USA) Petri dishes (Fischer Scientific), and cultured as described previously [16]. The SA culture was allowed to proliferate in a suspension of tryptic soy broth (TSB; Becton Dickinson) on a rotator at 37 °C until the optical density at 600 nm reached 0.1 (1 × 10^4^ CFU/ml) as measured by the spectrophotometer (Nanodrop 2000x UV-vis Spectrophotometer; Thermo Scientific). After MSC treatments, the media were removed and each well was washed three times with 0.5 ml of PBS and each well was replaced with fresh MSC media without minocycline. Immediately following, 20 μl of the SA suspension (200 CFU) was added to the MSC cultures and incubated at 37 °C for 6 hours. Cultures of MSC media alone and TSB alone with 200 CFU SA were included as controls. After 6 hours of culture, 50 μl of the coculture supernatant was removed and diluted serially (1:10) for colony forming ability enumeration as described previously [16]. Immediately following cocultures, supernatants were collected and spun at 12,000 × *g* for 10 minutes. Supernatants were removed from bacterial cell pellets and placed in a clean Eppendorf tube and stored at −20 °C until cytokine analysis. LIVE/DEAD® stain was applied to the MSCs remaining on the wells, quantified using a FLUOstar Omega plate reader, and imaged using a Nikon Eclipse TE300 microscope. Cytokine analysis of supernatants was performed using ELISA kits as already mentioned. Another experiment included MSC/SA coculture incubation with a mouse anti-human IL-6 antibody at 100 μg/ml (PeproTech, Rocky Hill, NJ, USA) during SA cocultures and were analyzed similarly [29]. MSCs were also cultured against SA in a transwell system to prevent cell-to-cell contact. MSCs were plated at 175,000 cells/well in the bottom of a six-well 0.4-μm transwell plate (Corning) with 2 ml of culture medium and cultured overnight. MSCs were then treated with or without minocycline for 24 hours and washed three times with PBS. SA was grown as already stated and 600 CFU (60 μl) of SA was added to 1 ml of culture medium in the top transwell and cocultured for 6 hours. After coculture, SA colony forming analysis was done as already stated and SA attachment to the top transwell surface was carried out using a gentian violet stain protocol as described previously [30].

### Internalization of *Staphylococcus aureus* by MSCs

MSCs were cultured overnight in six-well tissue culture plates (CellTreat) with glass coverslips added to the wells at 175,000 cells/well with 2 ml of MSC culture medium. MSC treatment with or without minocycline and SA culture preparation was performed as already stated, and then 60 μl of the SA suspension (600 CFU) was added to the MSC cultures and incubated at 37 °C for 4 hours. After coculture, media were removed and the wells were washed three times with PBS. Cells were fixed with 4% paraformaldehyde for 10 minutes, and then washed with PBS. Cells were then stained with Vacnomycin BOPIDY® FL Conjugate (ThermoFisher Scientific) at 1 μg/ml with ActinGreen™ 488 ReadyProbes™ (ThermoFisher Scientific) in PBS for 10 minutes at room temperature and washed with PBS. Coverslips were removed from the wells and stained with DAPI for 2 minutes, covered with a coverslip coated with VECTASHIELD® mounting medium, and imaged on an Olympus Fluoview FV1000. Total present SA and MSC-internalized SA were counted using ImageJ software (National Institutes of Health, MD, USA). MSCs were also cocultured in 48-well plates as already stated but including conditions with MSCs treated with minocycline in addition to the internalization inhibitors cholorquine (20 μM) and 3-methyladenine (5 mM) [31].

### Genetic expression analysis

MSCs were washed with PBS three times after MSC/SA cocultures in 48-well plates. MSC RNA was isolated by lysing MSCs in culture with 0.5 ml of TRIzol reagent (ThermoFisher Scientific) per well and the protocol was continued as described previously [17]. RT-qPCR was performed on synthesized cDNA using a StepOnePlus Real-Time PCR System (ThermoFisher Scientific) using TaqMan Expression Assay Probes for *IL-6* and *GAPDH*. Data pertaining to mRNAs were collected quantitatively and the CT cumber was corrected by CT readings of the corresponding internal control of *GAPDH*.

### In-vivo application of hydrogels in *Staphylococcus aureus*-inoculated full-thickness wounds

All animal experimental protocols were approved by the Institutional Animal Care and Use Committee (IACUC) of the University of Wisconsin-Madison. Female Sprague–Dawley rats (250–300 g, 12 weeks old) were grouped into three different cohorts (*n* = 3 rats per treatment type for each time point). Sprague–Dawley bone marrow-derived MSCs were similarly isolated, cultured, and characterized accordingly [26, 27]. MSC passages 4–6 were used for this in-vivo study. A day before the experiment, silicone O-rings (McMaster-Carr, Atlanta, GA, USA) were placed under ultraviolet light in a biosafety cabinet for at least 20 minutes and planktonic SA was grown in TSB to OD_600_ = 0.1 as stated earlier, aliquoted in 1 ml volumes in Eppendorf tubes, and frozen at −80 °C for overnight storage. On the day of the experiment, rat eyes were lubricated with Lubrifresh™ (Amazon, Seattle, WA, USA) and treated with buprenorphine (0.05 mg/kg). The rat dorsum was shaved and scrubbed with Betasept (4%; Amazon) and sterile saline. Two silicone O-rings were glued to the shaved dorsum of the rats using CrazyGlue Advanced Gel (Elmer’s Products Inc., Westerville, OH, USA) by firmly applying pressure for 2 minutes. The silicone O-rings were then sutured (Nylon 4.0; Ethicon, Sun Prairie, WI, USA) to inhibit healing by contraction, which is the accepted method for examining the healing of full-thickness wounds in rats [32]. Full-thickness wounds were created (two per rat) on the dorsum of the shaved rat using 8-mm biopsy punches (Miltex GmbH, York, PA, USA) while under isoflurane anesthesia (1.5% isoflurane, 1.5% oxygen). The wounds were imaged and then inoculated with 10 μl of thawed planktonic SA (OD_600_ = 0.1) and allowed to dry for 15 minutes. Hydrogels were formulated as described previously, applied to the wounds (100 μl), and subsequently polymerized as stated earlier with either: no antimicrobials or MSCs (no encapsulation); 1 × 10^6^ MSCs/ml (MSCs); or 1 × 10^6^ MSCs/ml + 50 μg/ml minocycline + 40 μg/ml vancomycin + 10 μg/ml linezolid (MSCs + mino. + vanco. + lin.) [17]. Hydrogels loaded with one or two drug combinations were not included to minimize the number of animals while maximizing the utility of the data obtained, and the direct effect of each entity promoting tissue healing via induction of MSC mechanisms was investigated in our proceeding in-vitro analysis. Each rat was randomly assigned to a treatment, with both wounds on the same animal receiving the same treatment. The treated wounds were allowed to dry for 2 minutes, then covered with Tegaderm™ film (3 M Healthcare, Neuss, Germany) and wrapped with cling gauze followed by Vetrap™ (3 M) tape. At 1 and 3 days after treatment application, rats were euthanized with Beuthanasia (0.2 ml/kg; Schering-Plough Animal Health Corp., Union, NJ, USA). The wounds were then imaged, before one wound bed per rat was harvested and put into 1 ml of sterile PBS with sterile beads for SA CFU enumeration, and one wound bed per rat was harvested, fixed in 10% neutral buffered formalin for 48 hours, paraffin embedded, and H&E stained [14, 33]. Wound beds collected for SA CFU enumeration were homogenized using a bullet blender (three 5-minute cycles; Next Advanced, Averill Park, NY, USA) and then diluted serially and cultured on agar plates for colony counting as already stated. Photomicrographs were taken of histological sections from H&E staining and the epidermal thickness of the healing wound tissue with clearly demarcated epidermal and dermal tissue boundaries was measured by averaging five epithelialized keratinocyte widths from randomized locations including the central region of the wound and areas adjacent to the wound margin.

### Statistical methods

All assays were conducted with at least three replicates per condition. Two-way ANOVA for multiple comparisons was used with a Bonferroni test assessment to determine *p* values for all assays with three or more conditions, and an unpaired two-tailed Student’s *t* test was used to determine *p* values for all assays with only two conditions. *p* < 0.05 was considered statistically significant. All analyses were performed with GraphPad Prism 5.0. Error bars represent the standard deviation of the specific group mean.

## Results

### Modulation of NFκB by minocycline in MSCs

**Fig. 1:**
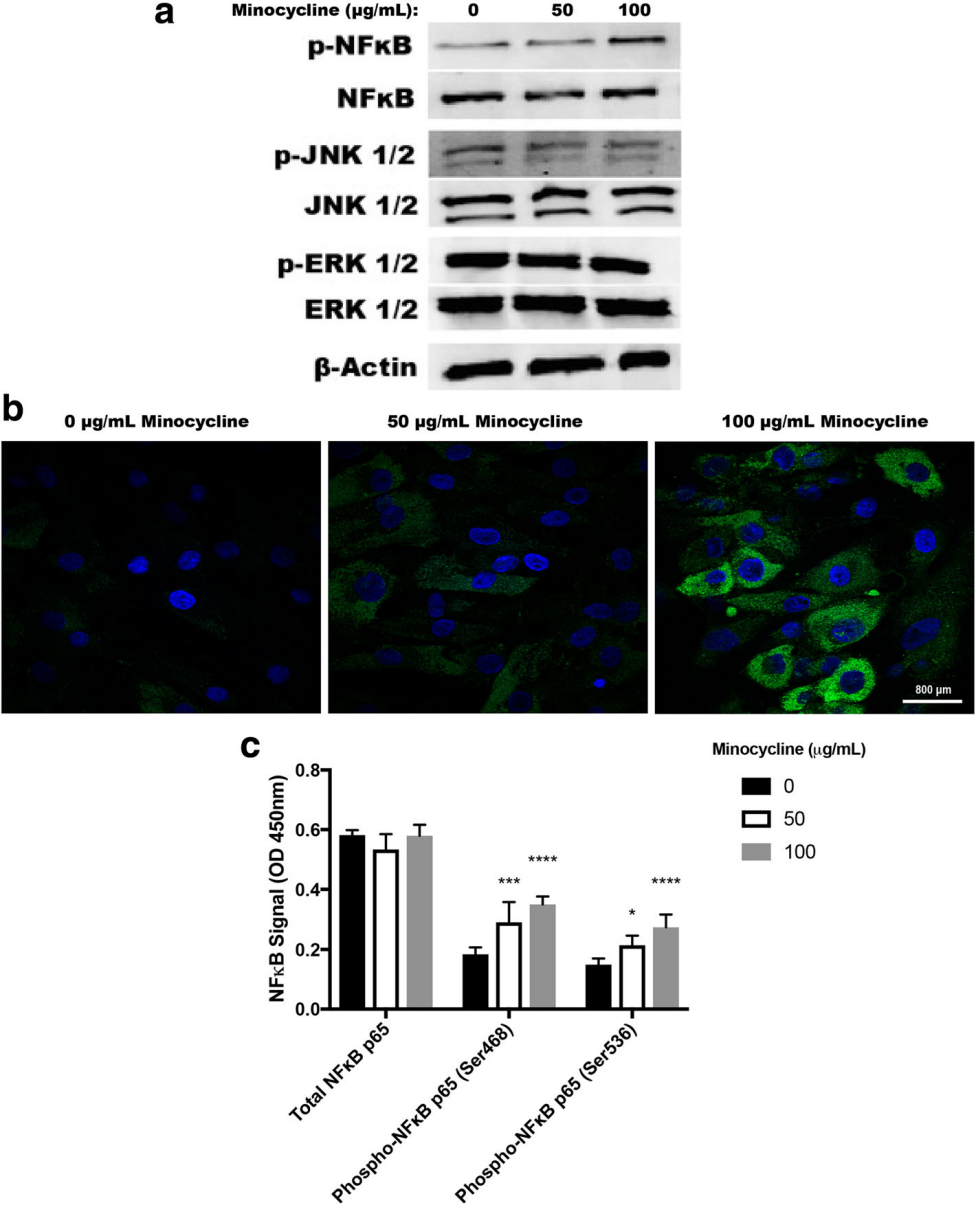
Phosphorylation of NFκB in MSCs by minocycline. **a** Western blot analysis of signaling protein phosphorylation by minocycline treatment in a dose-dependent manner. **b** Immunofluorescence of phosphorylated NFκB by minocycline in a dose-dependent manner (400× magnification, *scale bar* 800 μm). **c** Phosphorylation of specific NFκB amino acid residues by minocycline treatment in a dose-dependent manner. *p < 0.05, ***p < 0.005, ****p < 0.001. *OD* optical density

**Fig. 2:**
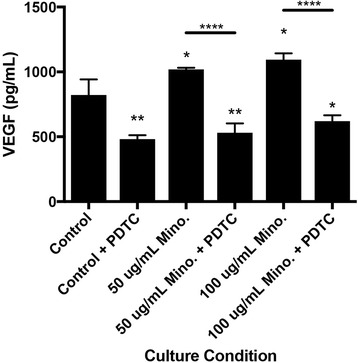
Production of VEGF by MSCs in the presence of minocycline and the NFκB inhibitor PDTC. *p < 0.05, **p < 0.01, ****p < 0.001. *PDTC* pyrrolidinedithiocarbamate ammonium, *VEGF* vascular endothelial growth factor

MSCs cultured with 100 μg/ml minocycline for 48 hours led to the presence of a phosphorylated NFκB western blot band from the protein extract that was not present in protein extracts derived from MSCs treated with 0 or 50 μg/ml minocycline for 48 hours. There were no differences in phosphorylation of JNK or ERK between all of the MSC culture conditions (Fig. 1a). Immunofluorescence shows that NFκB is phosphorylated by minocycline in a dose-dependent manner (Fig. 2a). NFκB is phosphorylated at the serine 468 residue when MSCs are treated with 50 μg/ml minocycline (*p* < 0.001 vs 0 μg/ml) and with 100 μg/ml minocycline (*p* < 0.0001 vs 0 μg/ml). NFκB is phosphorylated at the 536 residue when MSCs are treated with 50 μg/ml minocycline (*p* < 0.05 vs 0 μg/ml) and with 100 μg/ml minocycline (*p* < 0.0001 vs 0 μg/ml). Total levels of NFκB remained unchanged between treatment conditions (Fig. 1c). There was an increase in MSC proliferation after 24 hours of culture when MSCs were treated with 50 μg/ml minocycline (*p* < 0.0001 vs 0 μg/ml) and with 100 μg/ml minocycline (*p* < 0.0001 vs 0 μg/ml, *p* < 0.0001 vs 50 μg/ml) (Additional file 1: Figure S1).

### Effects of minocycline on VEGF and antimicrobial peptide LL-37 production

**Fig. 3:**
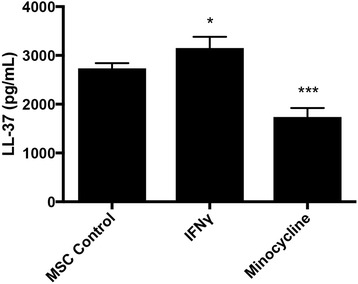
MSC production of the antibacterial peptide LL-37 from IFNγ or minocycline treatment. *p < 0.05, ***p < 0.005. *IFNγ* interferon gamma, *MSC* mesenchymal stromal/stem cell

There was an increase in VEGF production by MSCs when treated with 50 μg/ml minocycline (*p* < 0.05 vs 0 μg/ml) and with 100 μg/ml minocycline (*p* < 0.05 vs 0 μg/ml). The increased production of VEGF by minocycline was not present when MSCs were cultured with the NFκB inhibitor PDTC (control, *p* < 0.01; 50 μg/ml, *p* < 0.01; 100 μg/ml, *p* < 0.05) (Fig. 2). There was a decrease in LL-37 production in MSCs treated with 100 μg/ml minocycline for 24 hours (*p* < 0.001 vs MSC control), and there was an increase in LL-37 production in MSCs treated with 100 ng/ml IFN-γ for 24 hours (*p* < 0.05 vs MSC control) (Fig. 3).

### Minocycline effects on MSC antibacterial activity

**Fig. 4:**
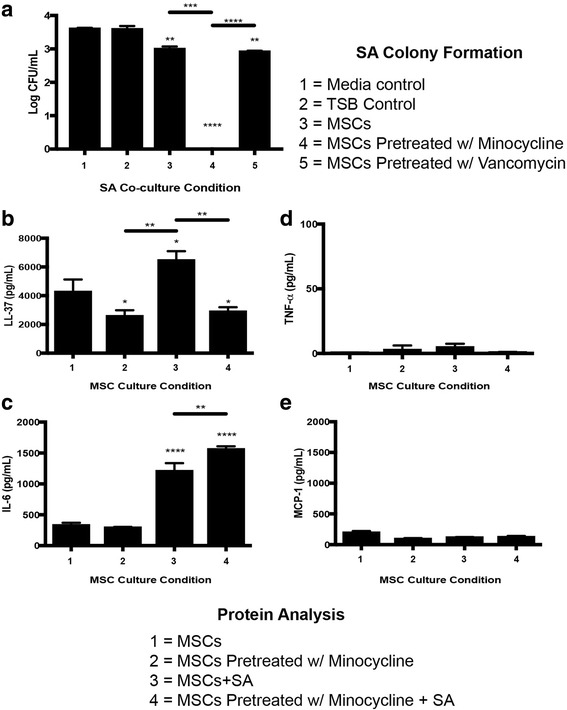
Antibacterial activity of MSCs against SA and subsequent supernatant cytokine analysis. **a** Colony forming abilities of SA after 6 hours of coculture with media controls, MSCs, or MSCs pretreated with antibiotics. Presence of antibacterial peptide LL-37 (**b**), IL-6 (**c**), TNF-α (**d**), and MCP-1 **e** in MSC/SA cocultures. *p < 0.05, **p < 0.01, ***p < 0.005, ****p < 0.001. *CFU* colony forming unit, *IL-6* interleukin-6, *LL-37* cathelicidin-related antimicrobial peptide, *MCP-1* monocyte chemotactic protein, *MSC* mesenchymal stromal/stem cell, *SA Staphylococcus aureus*, *TNF-α* tumor necrosis factor alpha, *TSB* tryptic soy broth

MSCs pretreated with 100 μg/ml minocycline for 24 hours demonstrated complete inhibition of SA colony forming abilities (*p* < 0.0001 vs media control), while MSCs not pretreated with minocycline or MSCs pretreated with 80 μg/ml vancomycin demonstrated a decrease in SA colony forming abilities (*p* < 0.01 vs MSC control). MSCs pretreated with minocycline demonstrated a significantly lower colony forming ability inhibition of SA than MSCs not pretreated with minocycline (*p* < 0.001) and MSCs pretreated with vancomycin (*p* < 0.0001) (Fig. 4a). MSCs not pretreated with minocycline demonstrated an increase in LL-37 production when cocultured with SA (*p* < 0.05 vs MSCs), while MSCs pretreated with 100 μg/ml minocycline demonstrated an inhibition of LL-37 production with and without SA coculture (*p* < 0.05 vs MSCs). MSCs not pretreated with minocycline and cocultured with SA produced higher levels of LL-37 compared to MSCs pretreated with minocycline without SA coculture (*p* < 0.01) and compared to MSCs pretreated with minocycline with SA coculture (*p* < 0.01) (Fig. 4b). Both MSCs not pretreated with minocycline and MSCs pretreated with minocycline demonstrated an increase in IL-6 production when cocultured with SA (*p* < 0.0001 vs MSCs), with minocycline-preconditioned MSCs demonstrating a higher production of IL-6 compared to MSCs not preconditioned with minocycline when both were cocultured with SA (*p* < 0.01) (Fig. 4c). MSCs not pretreated with minocycline and cocultured with SA demonstrated an increase in *IL-6* gene expression (*p* < 0.05 vs MSCs) and MSCs pretreated with minocycline and cocultured with SA demonstrated an increase in *IL-6* gene expression (*p* < 0.0001 vs MSCs), with minocycline-preconditioned MSCs demonstrating higher levels of *IL-6* gene expression compared to MSCs not preconditioned with minocycline when both were cocultured with SA (*p* < 0.001) (Additional file 2: Figure S2). SA colony forming abilities were higher when cocultured with MSCs pretreated with minocycline with anti-IL-6 present in cocultures compared to when cocultured with MSCs pretreated with minocycline without anti-IL-6 present in cocultures (*p* < 0.01) (Additional file 3: Figure S3). There were no differences in TNF-α or MCP-1 production levels between all MSC culture conditions (Fig. 4d, e).

**Fig. 5:**
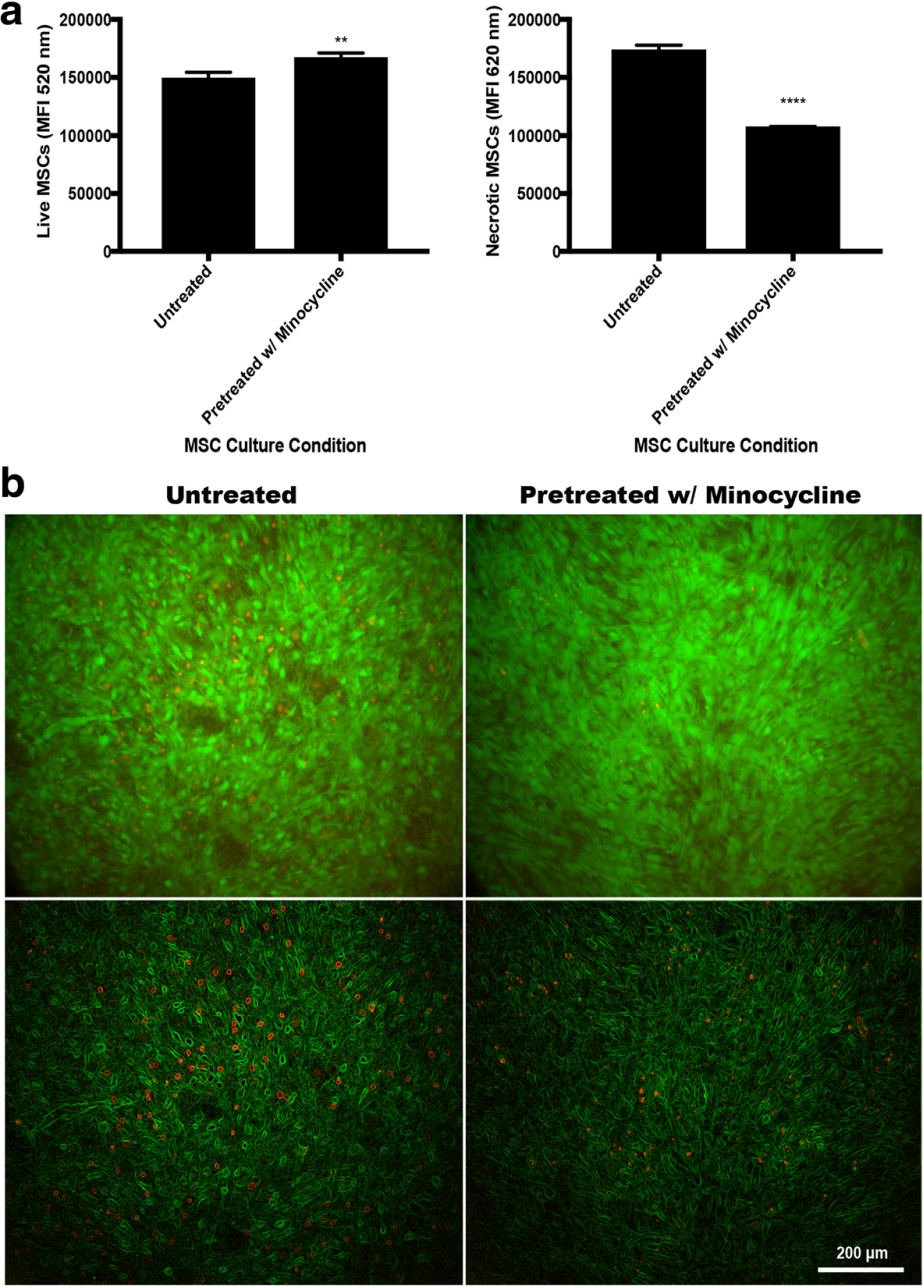
MSC viability after 6 hours of coculture with SA. **a** Live and necrotic fluorescence quantification of MSCs. **b** Fluorescent images indicating viable MSCs (*green*) and necrotic MSCs (*red*), with the bottom image only displaying cell borders (100× magnification, *scale bar* 200 μm). **p < 0.01, ****p < 0.001. *MSC* mesenchymal stromal/stem cell

**Fig. 6:**
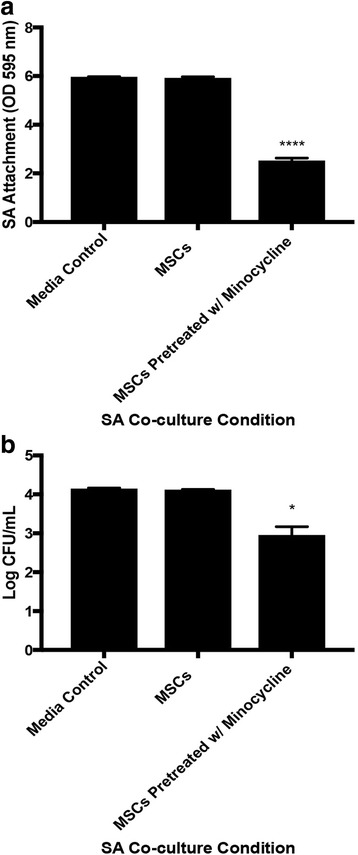
Antibacterial activity of MSCs against SA when cell-to-cell contact is prevented. **a** SA attachment to transwell surface. **b** Colony forming abilities of SA in transwell chamber. *p < 0.05, ****p < 0.001. *CFU* colony forming unit, *MSC* mesenchymal stromal/stem cell, *OD* optical density, *SA Staphylococcus aureus*

There was a higher level of viable MSCs after coculture with SA when pretreated with minocycline (*p* < 0.01 vs untreated) and there was a lower level of necrotic MSCs after coculture with SA when pretreated with minocycline (*p* < 0.0001 vs untreated) (Fig. 5a). Live/dead images with only cell borders visible suggest a higher presence of necrotic MSCs in the untreated condition (Fig. 5b). Attachment of SA to the transwell surface in transwell cocultures was decreased when cocultured with MSCs pretreated with minocycline (*p* < 0.0001 vs media control) and SA colony forming abilities were lowered, but not absent, in transwell cocultures when cocultured with MSCs pretreated with 100 μg/ml minocycline (*p* < 0.05 vs media control) (Fig. 6a, b).

**Fig. 7:**
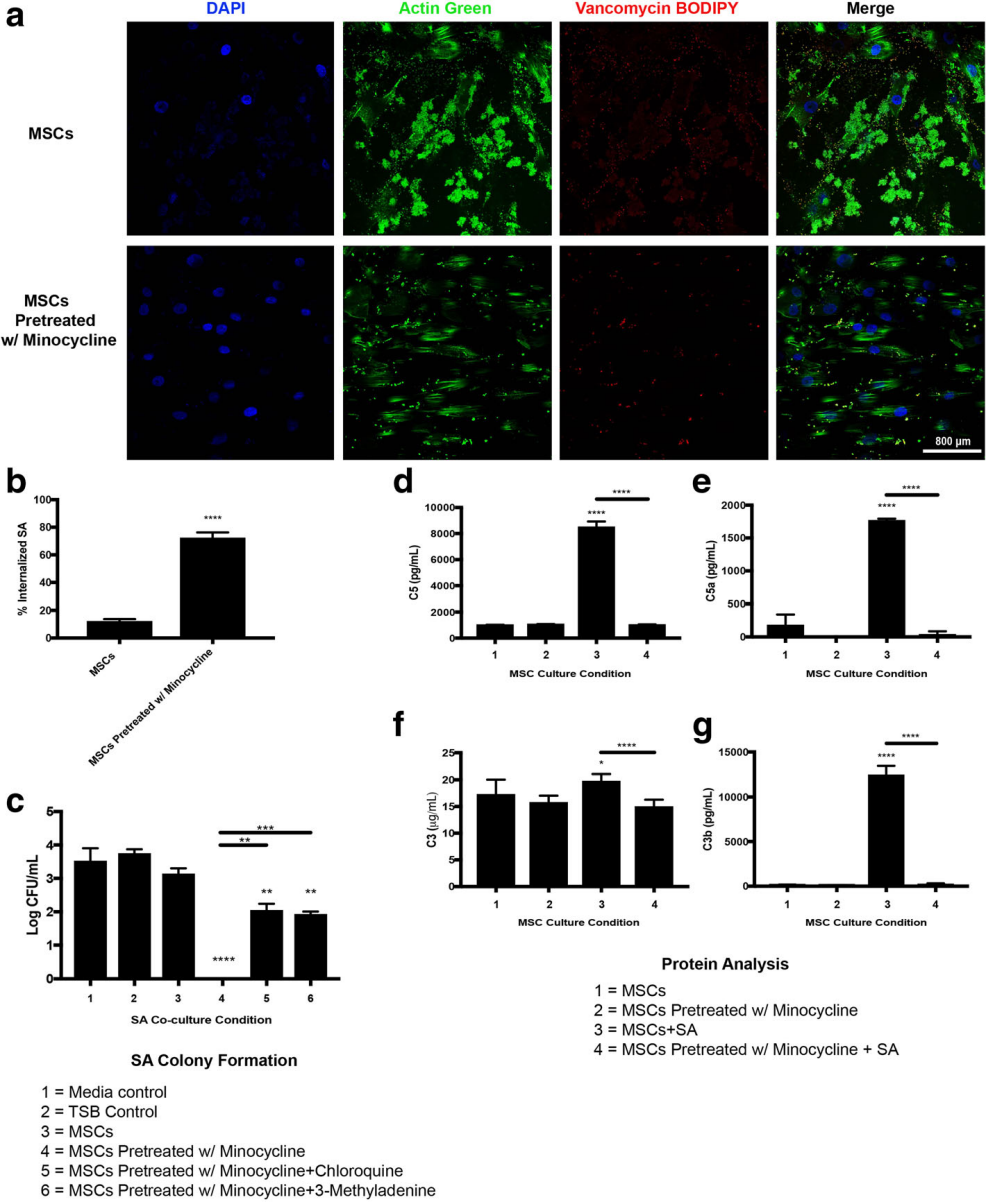
Internalization of SA by MSCs and complement system activation. **a** Confocal images of SA internalization by MSCs after 4 hours of MSC and SA coculture on glass coverslips indicating MSC nuclei (DAPI), MSC cell bodies (actin green) and SA (vancomycin BODIPY) (400× magnification, *scale bar* 800 μm). **b** Quantification of SA internalization by MSCs. **c** Colony forming abilities of SA after 6 hours of cocultures including MSC treatments with minocycline and internalization inhibitors chloroquine or 3-methyladenine. C5 (**d**), C5a (**e**), C3 (**f**), and C3b (**g**) protein levels in MSC and SA cocultures. *p < 0.05, **p < 0.01, ***p < 0.005, ****p < 0.001. *CFU* colony forming unit, *MSC* mesenchymal stromal/stem cell, *SA Staphylococcus aureus*, *TNF-α* tumor necrosis factor alpha, *TSB* tryptic soy broth

MSCs demonstrated a higher level of SA internalization when pretreated with 100 μg/ml minocycline (*p* < 0.0001 vs MSCs) (Fig. 7a, b). SA colony forming abilities were higher in cocultures with MSCs pretreated with minocycline when internalization inhibitor cholorquine (*p* < 0.01 vs MSCs pretreated with minocycline) or 3-methyladenine (*p* < 0.001 vs MSCs pretreated with minocycline) was included in the minocycline pretreatment. SA colony forming abilities were still lower in cocultures with MSCs pretreated with minocycline and chloroquine (*p* < 0.01 vs media control) and in cocultures with MSCs pretreated with minocycline and 3-methyladenine (*p* < 0.01 vs media control) (Fig. 7c). Production of complement protein C5 was higher in MSCs cocultured with SA when not pretreated with minocycline (*p* < 0.0001 vs MSCs, *p* < 0.0001 vs MSCs pretreated with minocycline + SA). Production of the cleaved complement protein C5a was higher in MSCs cocultured with SA when not pretreated with minocycline (*p* < 0.0001 vs MSCs, *p* < 0.0001 vs MSCs pretreated with minocycline + SA). Production of the complement protein C3 was higher in MSCs cocultured with SA when not pretreated with minocycline (*p* < 0.05 vs MSCs, *p* < 0.0001 vs MSCs pretreated with minocycline + SA). Production of the cleaved complement protein C3b was higher in MSCs cocultured with SA when not pretreated with minocycline (*p* < 0.0001 vs MSCs, *p* < 0.0001 vs MSCs pretreated with minocycline + SA) (Fig. 7d–g).

### In-vivo antimicrobial efficacy of MSC and antibiotic-loaded hydrogels

**Fig. 8:**
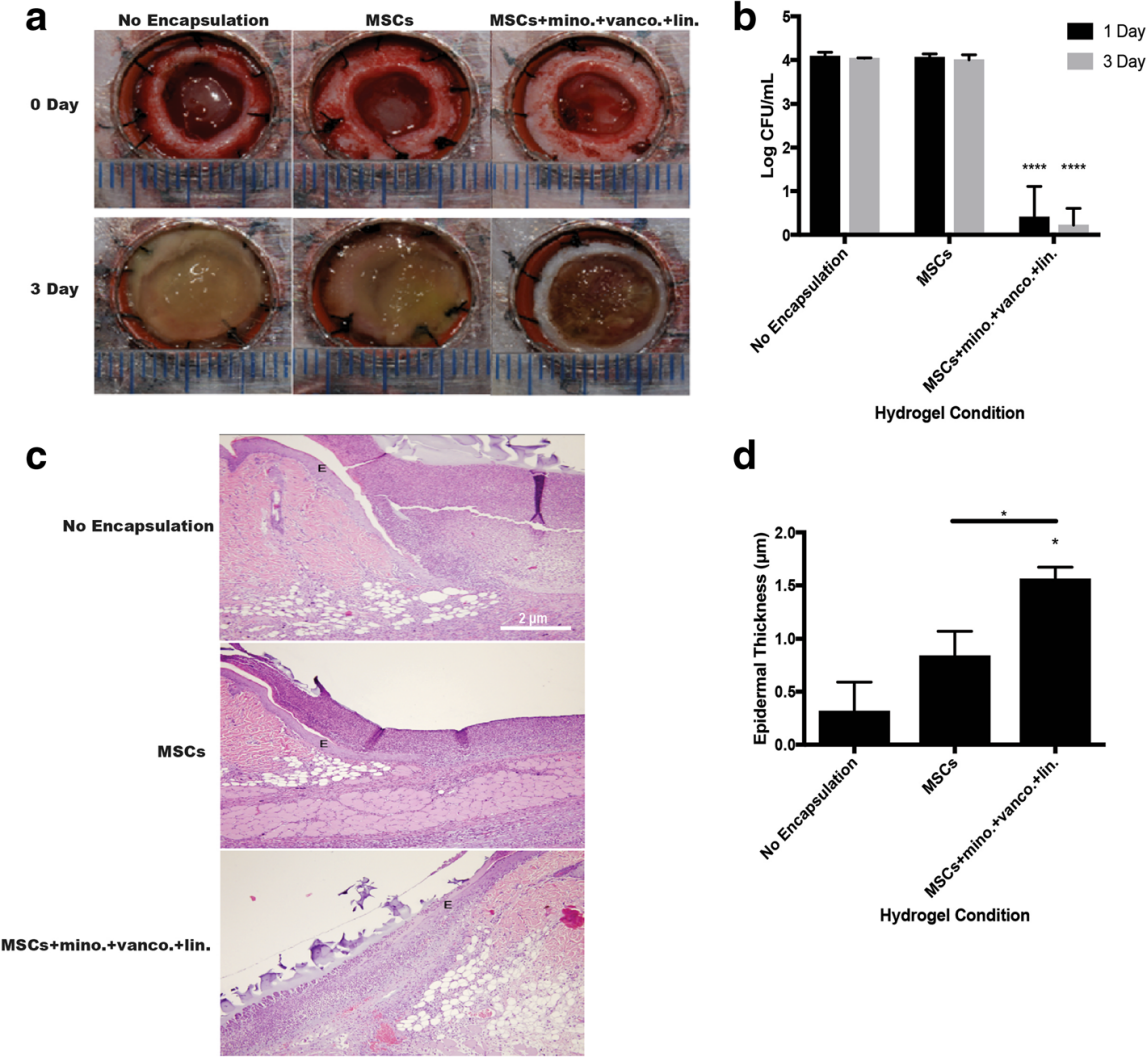
In-vivo antimicrobial efficacy of antimicrobial-loaded hydrogels in SA-inoculated wounds with histological analysis. **a** Wound bed images of SA-inoculated full-thickness cutaneous wounds (*ticks* millimeter marks) before hydrogel treatment and 3 days after hydrogel treatment. **b** SA colony forming abilities after 1 and 3 days in the wound bed with hydrogels. **c**. H&E representative full-thickness wound images of one side of the wound (100× magnification, *scale bar* 2 μm, *E* epithelial layer). **d** Epidermal thickness in wounds 3 days after hydrogel treatment. *p < 0.05, ****p < 0.001. *CFU* colony forming unit, *MSC* mesenchymal stromal/stem cell, *MSCs + mino. + vanco. + lin.* 1 × 10^6^ MSCs/ml + 50 μg/ml minocycline + 40 μg/ml vancomycin + 10 μg/ml linezolid

The presence of bacteria and colony forming abilities of SA in the wound beds were significantly lower after 1 day (*p* < 0.0001 vs no encapsulation) and 3 days (*p* < 0.0001 vs no encapsulation) of treatment (Fig. 8a, b). Epidermal thickness of wounds treated with MSC and antibiotic-loaded hydrogels was higher (*p* < 0.05 vs no encapsulation, *p* < 0.05 vs MSCs) after 3 days of treatment.

## Discussion

Small molecules are known to influence the immunomodulatory and antibacterial properties of MSCs. Adipose tissue-derived MSCs can be treated with rapamycin which leads to the production of anti-inflammatory molecules that could help prevent acute graft-versus-host disease (aGVHD) [34]. Menstrual-derived MSCs have shown a synergistic antimicrobial effect with antibiotics in improving survival rates and enhancing bacterial clearance with reduced organ injury in an animal sepsis model [35]. Minocycline has an immunomodulatory effect on bone marrow-derived MSCs that leads to a synergistic effect with minocycline itself in treating autoimmune encephalomyelitis [36]. Bone marrow-derived MSCs have therapeutic benefits in other multiple applications where the effects of antibiotic treatment are unknown, and it would be beneficial to investigate the effects of minocycline treatment on these applications. For example, MSCs increase the thickness of the ventricular wall in a heart failure model, and they improve cardiac function with enhanced ventricular remodeling and reduced cardiac fibrosis in a heart transplantation model [37, 38]. This effect could be influenced by a minocycline-induced production of VEGF [17]. MSCs can also influence macrophage polarization, and MSCs have been shown to increase the frequency of alternatively activated macrophages in the heart after acute myocardial infarction [39]. The effect of minocycline-treated MSCs on macrophage polarization would be a logical future study to investigate further immunomodulatory effects of minocycline-treated MSCs [40, 41].

We have shown previously that minocycline can enhance the wound healing properties in human bone marrow MSCs, but the mechanism of enhancement was still unknown [17]. Here, we demonstrate that this enhancement of the wound healing phenotype may be due to the phosphorylation and potential activation of NFκB in MSCs. NFκB is a transcription factor that affects gene expression changes in responses to stimuli including cytokines and bacterial antigens that can ultimately regulate immune responses to infection [42, 43]. NFκB controls the expression of inducible chemokines, cell adhesion molecules, and vasoactive and anti-apoptotic proteins [44–46]. Activation of the NFκB pathway in MSCs due to stimulation by TNF-α, LPS, or hypoxia has led to the increased production of multiple growth factors by MSCs including VEGF, FGF2, IGF-1, and HGF that could play a crucial role in the wound healing attributes of MSCs [21]. Activation of the NFκB pathway though the TNF receptor family can lead to the cytoplasmic phosphorylation of NFκB at serine 536, as observed in this study, that leads to IκB kinase ε (IKKε) translocation to the nucleus, where it can associate with the promoter regions of several NFκB target genes and phosphorylate other transcription factors, ultimately leading to the transcription of a subset of NFκB target genes [47]. Activation of the NFκB pathway in MSCs due to stimulation by TNF-α has also shown to enhance IL-6, vascular cell adhesion molecule-1 expression (VCAM-1), and intercellular adhesion molecule 1 (ICAM-1) production [18, 47, 48]. These studies suggest that the NFκB pathway may be activated through the interaction of minocycline with the TNF receptor family which leads to the recruitment of an IκB kinase (IKK) that phosphorylates inhibitor of kappa B (IκB) on NFκB and ultimately leads to IκB polyubiquitination and degradation by the proteasome which allows for cytoplasmic p65 phosphorylation and downstream effects [49]. This mechanism of NFκB cytoplasmic phosphorylation and IKKε nuclear translocation may be responsible for the minocycline-induced enhancement of VEGF and IL-6 production, as well as enhanced adhesion molecule production in MSCs as we observed previously. Indeed, this is suggested by inhibiting the NFκB pathway in combination with minocycline treatment that ultimately prevented the minocycline-induced increase in MSC VEGF production [17].

In addition to NFκB phosphorylation, treatment of MSCs with the antibacterial peptide LL-37 increases MSC migration, proliferation, growth factor expression, and tissue regeneration capacity similar to what we observed in our previous study [17, 24]. Interestingly, LL-37 activates the NFκB pathway in embryonic endothelial progenitor cells that enhances recruitment to muscle tissue and in periodontal ligament cells that led to enhanced VEGF production [50, 51]. MSCs produce LL-37 in response to infection that leads to antibacterial activity against *E. coli*, *Psudomonas aeruginosa*, and SA [9]. LL-37 produced by MSCs has been shown to be synergistic with the aminoglycoside antibiotic geneticin in a cystic fibrosis infection treatment model and decreased the severity of lung injury in *E. coli*-infected lungs [10, 52]. However, our results show that minocycline actually inhibits the production of LL-37 in MSCs both alone and when stimulated by SA, suggesting that the NFκB pathway is activated by minocycline itself and not from an induction of LL-37 production. Because LL-37 has been proven to be a vital peptide in MSC antibacterial activity, we determined whether minocycline treatment had an effect on MSC antibacterial activity against SA.

Interestingly, pretreatment of MSCs with minocycline leads to greatly enhanced antibacterial activity against SA in which no colony formation was observed after coculture. MSC IL-6 production is increased in MSCs cocultured with SA, but both production and gene expression of IL-6 was higher in MSCs after SA coculture when pretreated with minocycline. NFκB activation through lipopolysaccharide (LPS) stimulation has been shown to control IL-6 production in macrophages, and an increase in IL-6 through NFκB activation was not accompanied by an increase in TNF-α [53] as we also observed in this study. IL-6 stimulates antibacterial responses of immune regulating cells and has been shown to initiate antibacterial activity of keratinocyte grafts against SA through an increase in the synthesis of cytokines that could be cytotoxic to SA [54]. Although we demonstrate that IL-6 is a major factor in the minocycline-enhanced antibacterial activity of MSCs, it is not the only factor, because reduction of IL-6 with an antibody does not lead to recovery of the full colony forming ability of SA. Previous studies suggest that phagocytosis of SA may be a part of the MSC antibacterial profile [55, 56].

Separating physical contact of MSCs and SA through a transwell system led to the formation of SA colonies when cocultured with MSCs pretreated with minocycline, suggesting that cell-to-cell contact is necessary for optimal antibacterial activity of MSCs against SA. There are multiple ways in which MSCs can demonstrate antibacterial activity through cell-to-cell contact. MSC immunoregulation includes activation of the complement system proteins, which is generally suppressed by MSC production of factor H but can be induced through inflammatory factors that may be secreted by SA [57]. The activation of the NFκB pathway is correlated with the cleavage of complement protein C5 to C5a and C5b in MSCs that leads to a cascade which forms a Membrane Attack Complex (MAC) that induces cell death in microbes [58, 59]. However, in this study we see that minocycline inhibits both production of C5 and the cleavage of C5 into C5a. The complement protein C3 is produced and cleaved into the active C3b protein by MSCs as well, which leads to opsonization that ultimately leads to bacterial phagocytosis [60, 61]. Again, minocycline inhibits C3 production and cleavage into C3b in MSCs, suggesting the cell-to-cell contact antibacterial activity is accomplished by different means. Because the complement system seems to be suppressed in MSCs by pretreatment with minocycline, it is possible that minocycline induces the production of factor H even in the presence of inflammatory factors produced by SA [57]. Bacterial internalization and an increase in IL-6 production are strongly correlated [55, 62]. The increased internalization of SA by MSCs may have been due to NFκB activation of minocycline, which increased IL-6 production in the presence of SA that ultimately induced the increased SA internalization, or the potential preservation of MSC viability by minocycline pretreatment may have allowed MSCs to carry on normal internalization activity.

Vancomycin and linezolid have different mechanisms of antibacterial activity against SA than minocycline, and are not known to modulate NFκB in mammalian cells [63–65]. Additionally, minocycline has been shown to modulate neuroinflammation independently of its antimicrobial activity in brain abscesses caused by SA infection, suggesting minocycline’s antimicrobial and immunomodulatory enhancement effects on other cell types [66]. The specific minocycline-induced antimicrobial enhancement of MSCs may have contributed to the in-vivo antimicrobial efficacy of MSC and triple antibiotic-loaded hydrogels that reduced SA bioburden and increased reepithelialization in SA-inoculated wounds.

## Conclusions

Minocycline modulates the NFκB pathway and enhances antibacterial activity against SA via an increase in IL-6 production and SA internalization by MSCs. MSC-loaded hydrogels containing minocycline reduce bacterial bioburden in SA-inoculated full-thickness cutaneous wounds.

## Additional files

Additional file 1: Figure S1. Showing proliferation of MSCs after 24 hours of minocycline treatment in a dose-dependent manner. (JPG 103 kb)

Additional file 2: Figure S2. Showing expression of *IL6* in MSCs when cultured alone or with SA with and without minocycline pretreatment. (JPG 171 kb)

Additional file 3: Figure S3. Showing colony forming abilities of SA after 6 hours of coculture with media controls, MSCs with or without anti-IL-6, and MSCs pretreated with minocycline with or without anti-IL-6. (JPG 146 kb)

## Abbreviations

aGVHD: Acute graft-versus-host disease
ANOVA: Analysis of variance
BSA: Bovine serum albumin
cDNA: Complementary deoxyribonucleic acid
CFU: Colony forming unit
CT: Cycle threshold
ECM: Extracellular matrix
ELISA: Enzyme-linked immuunosorbent assay
FGF2: Fibroblast growth factor 2
GAPDH: Glyceraldehyde 3-phosphate dehydrogenase
H&E: Hematoxylin and eosin
HGF: Hepatocyte growth factor
ICAM-1: Intercellular adhesion molecule 1
IFNγ: Interferon gamma
IGF-1: Insulin-like growth factor 1
IgG: Immunoglobulin G
IKK: IκB kinase
IKKε: IκB kinase ε
IL-6: Interleukin-6
IκB: Inhibitor of kappa B
JNK/SAPK: c-Jun NH_2_-terminal kinase
LL-37: Cathelicidin-related antimicrobial peptide
LPS: Lipopolysaccharide
MAPK/ERK: Mitogen-activated protein kinase
MCP-1: Monocyte chemotactic protein
MSC: Mesenchymal stromal/stem cell
NFκB: Transcriptional nuclear factor-κB
OD: Optical density
PBS: Phosphate-buffered saline
PDTC: Pyrrolidinedithiocarbamate ammonium
RT-qPCR: Real-time quantitative polymerase chain reaction
SA: *Staphylococcus aureus*
TBS: Tris-buffered saline
TNF-α: Tumor necrosis factor alpha
TSB: Tryptic soy broth
VCAM-1: Vascular cell adhesion molecule-1
VEGF: Vascular endothelial growth factor

## References

[1] Caplan AI (2009). Why are MSCs therapeutic? New data: new insight. J Pathol.

[2] Reitamo S, Remitz A, Tamai K, Ledo I, Uitto J (1994). Interleukin 10 up-regulates elastin gene expression in vivo and in vitro at the transcriptional level. Biochem J.

[3] Jeon YK, Jang YH, Yoo DR, Kim SN, Lee SK, Nam MJ (2010). Mesenchymal stem cells’ interaction with skin: wound-healing effect on fibroblast cells and skin tissue. Wound Repair Regen.

[4] Brown JM, Nemeth K, Kushnir-Sukhov NM, Metcalfe DD, Mezey E (2011). Bone marrow stromal cells inhibit mast cell function via a COX2-dependent mechanism. Clin Exp Allergy.

[5] Renault MA, Roncalli J, Tongers J, Misener S, Thorne T, Jujo K, Ito A, Clarke T, Fung C, Millay M (2009). The Hedgehog transcription factor Gli3 modulates angiogenesis. Circ Res.

[6] Gruber R, Kandler B, Holzmann P, Vögele-Kadletz M, Losert U, Fischer MB, Watzek G (2005). Bone marrow stromal cells can provide a local environment that favors migration and formation of tubular structures of endothelial cells. Tissue Eng.

[7] Kaigler D, Krebsbach PH, Polverini PJ, Mooney DJ (2003). Role of vascular endothelial growth factor in bone marrow stromal cell modulation of endothelial cells. Tissue Eng.

[8] Au P, Tam J, Fukumura D, Jain RK (2008). Bone marrow-derived mesenchymal stem cells facilitate engineering of long-lasting functional vasculature. Blood.

[9] Krasnodembskaya A, Song Y, Fang X, Gupta N, Serikov V, Lee JW, Matthay MA (2010). Antibacterial effect of human mesenchymal stem cells is mediated in part from secretion of the antimicrobial peptide LL-37. Stem Cells.

[10] Sutton MT, Fletcher D, Ghosh SK, Weinberg A, van Heeckeren R, Kaur S, Sadeghi Z, Hijaz A, Reese J, Lazarus HM (2016). Antimicrobial properties of mesenchymal stem cells: therapeutic potential for cystic fibrosis infection, and treatment. Stem Cells Int.

[11] Fu Y, Xu K, Zheng X, Giacomin AJ, Mix AW, Kao WJ (2012). 3D cell entrapment in crosslinked thiolated gelatin-poly(ethylene glycol) diacrylate hydrogels. Biomaterials.

[12] Cantu DA, Hematti P, Kao WJ (2012). Cell encapsulating biomaterial regulates mesenchymal stromal/stem cell differentiation and macrophage immunophenotype. Stem Cells Transl Med.

[13] Wang C, Varshney RR, Wang DA (2010). Therapeutic cell delivery and fate control in hydrogels and hydrogel hybrids. Adv Drug Deliv Rev.

[14] Xu K, Cantu DA, Fu Y, Kim J, Zheng X, Hematti P, Kao WJ (2013). Thiol-ene Michael-type formation of gelatin/poly(ethylene glycol) biomatrices for three-dimensional mesenchymal stromal/stem cell administration to cutaneous wounds. Acta Biomater.

[15] Lin H, Yang G, Tan J, Tuan RS (2012). Influence of decellularized matrix derived from human mesenchymal stem cells on their proliferation, migration and multi-lineage differentiation potential. Biomaterials.

[16] Guerra AD, Cantu DA, Vecchi JT, Rose WE, Hematti P, Kao WJ (2015). Mesenchymal stromal/stem cell and minocycline-loaded hydrogels inhibit the growth of Staphylococcus aureus that evades immunomodulation of blood-derived leukocytes. AAPS J.

[17] Daniel Guerra A, Rose WE, Hematti P, John Kao W (2017). Minocycline enhances the mesenchymal stromal/stem cell pro-healing phenotype in triple antimicrobial-loaded hydrogels. Acta Biomater.

[18] Lu ZY, Chen WC, Li YH, Li L, Zhang H, Pang Y, Xiao ZF, Xiao HW, Xiao Y (2016). TNF-α enhances vascular cell adhesion molecule-1 expression in human bone marrow mesenchymal stem cells via the NF-κB, ERK and JNK signaling pathways. Mol Med Rep.

[19] Zhong X, Li X, Liu F, Tan H, Shang D (2012). Omentin inhibits TNF-α-induced expression of adhesion molecules in endothelial cells via ERK/NF-κB pathway. Biochem Biophys Res Commun.

[20] Choi H, Nguyen HN, Lamb FS (2014). Inhibition of endocytosis exacerbates TNF-α-induced endothelial dysfunction via enhanced JNK and p38 activation. Am J Physiol Heart Circ Physiol.

[21] Crisostomo PR, Wang Y, Markel TA, Wang M, Lahm T, Meldrum DR (2008). Human mesenchymal stem cells stimulated by TNF-alpha, LPS, or hypoxia produce growth factors by an NF kappa B- but not JNK-dependent mechanism. Am J Physiol Cell Physiol.

[22] Jarrar D, Kuebler JF, Rue LW, Matalon S, Wang P, Bland KI, Chaudry IH (2002). Alveolar macrophage activation after trauma-hemorrhage and sepsis is dependent on NF-kappaB and MAPK/ERK mechanisms. Am J Physiol Lung Cell Mol Physiol.

[23] Wang M, Crisostomo PR, Herring C, Meldrum KK, Meldrum DR (2006). Human progenitor cells from bone marrow or adipose tissue produce VEGF, HGF, and IGF-I in response to TNF by a p38 MAPK-dependent mechanism. Am J Physiol Regul Integr Comp Physiol.

[24] Yang Y, Choi H, Seon M, Cho D, Bang SI (2016). LL-37 stimulates the functions of adipose-derived stromal/stem cells via early growth response 1 and the MAPK pathway. Stem Cell Res Ther.

[25] Coffelt SB, Marini FC, Watson K, Zwezdaryk KJ, Dembinski JL, LaMarca HL, Tomchuck SL, Honer zu Bentrup K, Danka ES, Henkle SL, Scandurro AB (2009). The pro-inflammatory peptide LL-37 promotes ovarian tumor progression through recruitment of multipotent mesenchymal stromal cells. Proc Natl Acad Sci U S A.

[26] Trivedi P, Hematti P (2008). Derivation and immunological characterization of mesenchymal stromal cells from human embryonic stem cells. Exp Hematol.

[27] Dominici M, Le Blanc K, Mueller I, Slaper-Cortenbach I, Marini F, Krause D, Deans R, Keating A, Prockop D, Horwitz E (2006). Minimal criteria for defining multipotent mesenchymal stromal cells. The International Society for Cellular Therapy position statement. Cytotherapy.

[28] Ihry RJ, Sapiro AL, Bashirullah A (2012). Translational control by the DEAD Box RNA helicase belle regulates ecdysone-triggered transcriptional cascades. PLoS Genet.

[29] Cortini M, Massa A, Avnet S, Bonuccelli G, Baldini N (2016). Tumor-activated mesenchymal stromal cells promote osteosarcoma stemness and migratory potential via IL-6 secretion. PLoS One.

[30] Kiran MD, Adikesavan NV, Cirioni O, Giacometti A, Silvestri C, Scalise G, Ghiselli R, Saba V, Orlando F, Shoham M, Balaban N (2008). Discovery of a quorum-sensing inhibitor of drug-resistant staphylococcal infections by structure-based virtual screening. Mol Pharmacol.

[31] Song BQ, Chi Y, Li X, Du WJ, Han ZB, Tian JJ, Li JJ, Chen F, Wu HH, Han LX (2015). Inhibition of Notch signaling promotes the adipogenic differentiation of mesenchymal stem cells through autophagy activation and PTEN-PI3K/AKT/mTOR pathway. Cell Physiol Biochem.

[32] Dorsett-Martin WA (2004). Rat models of skin wound healing: a review. Wound Repair Regen.

[33] Agarwal A, Nelson TB, Kierski PR, Schurr MJ, Murphy CJ, Czuprynski CJ, McAnulty JF, Abbott NL (2012). Polymeric multilayers that localize the release of chlorhexidine from biologic wound dressings. Biomaterials.

[34] Kim KW, Moon SJ, Park MJ, Kim BM, Kim EK, Lee SH, Lee EJ, Chung BH, Yang CW, Cho ML (2015). Optimization of adipose tissue-derived mesenchymal stem cells by rapamycin in a murine model of acute graft-versus-host disease. Stem Cell Res Ther.

[35] Alcayaga-Miranda F, Cuenca J, Martin A, Contreras L, Figueroa FE, Khoury M (2015). Combination therapy of menstrual derived mesenchymal stem cells and antibiotics ameliorates survival in sepsis. Stem Cell Res Ther.

[36] Hou Y, Ryu CH, Park KY, Kim SM, Jeong CH, Jeun SS (2013). Effective combination of human bone marrow mesenchymal stem cells and minocycline in experimental autoimmune encephalomyelitis mice. Stem Cell Res Ther.

[37] Dayan V, Yannarelli G, Filomeno P, Keating A (2012). Human mesenchymal stromal cells improve scar thickness without enhancing cardiac function in a chronic ischaemic heart failure model. Interact Cardiovasc Thorac Surg.

[38] Montanari S, Dayan V, Yannarelli G, Billia F, Viswanathan S, Connelly KA, Keating A (2015). Mesenchymal stromal cells improve cardiac function and left ventricular remodeling in a heart transplantation model. J Heart Lung Transplant.

[39] Dayan V, Yannarelli G, Billia F, Filomeno P, Wang XH, Davies JE, Keating A (2011). Mesenchymal stromal cells mediate a switch to alternatively activated monocytes/macrophages after acute myocardial infarction. Basic Res Cardiol.

[40] Kim L, Hematti P (2009). Mesenchymal stem cell-educated macrophages: a novel type of alternatively activated macrophages. Exp Hematol.

[41] Bouchlaka MN, Moffitt AB, Kim J, Kink JA, Bloom DD, Love C, Dave S, Hematti P, Capitini CM (2017). Human mesenchymal stem cell-educated macrophages are a distinct high IL-6-producing subset that confer protection in graft-versus-host-disease and radiation injury models. Biol Blood Marrow Transplant.

[42] Gilmore TD (2006). Introduction to NF-kappaB: players, pathways, perspectives. Oncogene.

[43] Brasier AR (2006). The NF-kappaB regulatory network. Cardiovasc Toxicol.

[44] Pahl HL (1999). Activators and target genes of Rel/NF-kappaB transcription factors. Oncogene.

[45] Tian B, Nowak DE, Brasier AR (2005). A TNF-induced gene expression program under oscillatory NF-kappaB control. BMC Genomics.

[46] Tian B, Nowak DE, Jamaluddin M, Wang S, Brasier AR (2005). Identification of direct genomic targets downstream of the nuclear factor-kappaB transcription factor mediating tumor necrosis factor signaling. J Biol Chem.

[47] Moreno R, Sobotzik J, Schultz C, Schmitz ML (2010). Specification of the NF-κB transcriptional response by p65 phosphorylation and TNF-induced nuclear translocation of IKKε. Nucleic Acids Res.

[48] Eliopoulos AG, Stack M, Dawson CW, Kaye KM, Hodgkin L, Sihota S, Rowe M, Young LS (1997). Epstien-Barr virus-encoded LMP1 and CD40 mediate IL-6 production in epithelial cells via an NF-κB pathway involving TNF receptor-associated factors. Oncogene.

[49] Liu ZG (2005). Molecular mechanism of TNF signaling and beyond. Cell Res.

[50] Pfosser A, El-Aouni C, Pfisterer I, Dietz M, Globisch F, Stachel G, Trenkwalder T, Pinkenburg O, Horstkotte J, Hinkel R (2010). NF kappaB activation in embryonic endothelial progenitor cells enhances neovascularization via PSGL-1 mediated recruitment: novel role for LL37. Stem Cells.

[51] Kittaka M, Shiba H, Kajiya M, Ouhara K, Takeda K, Kanbara K, Fujita T, Kawaguchi H, Komatsuzawa H, Kurihara H (2013). Antimicrobial peptide LL37 promotes vascular endothelial growth factor-A expression in human periodontal ligament cells. J Periodontal Res.

[52] Devaney J, Horie S, Masterson C, Elliman S, Barry F, O’Brien T, Curley GF, O’Toole D, Laffey JG (2015). Human mesenchymal stromal cells decrease the severity of acute lung injury induced by E. coli in the rat. Thorax.

[53] Sárvári AK, Doan-Xuan QM, Bacsó Z, Csomós I, Balajthy Z, Fésüs L (2015). Interaction of differentiated human adipocytes with macrophages leads to trogocytosis and selective IL-6 secretion. Cell Death Dis.

[54] Erdag G, Morgan JR (2002). Interleukin-1alpha and interleukin-6 enhance the antibacterial properties of cultured composite keratinocyte grafts. Ann Surg.

[55] Josse J, Velard F, Mechiche Alami S, Brun V, Guillaume C, Kerdjoudj H, Lamkhioued B, Gangloff SC (2014). Increased internalization of Staphylococcus aureus and cytokine expression in human Wharton’s jelly mesenchymal stem cells. Biomed Mater Eng.

[56] Kriebel K, Biedermann A, Kreikemeyer B, Lang H (2013). Anaerobic co-culture of mesenchymal stem cells and anaerobic pathogens—a new in vitro model system. PLoS One.

[57] Tu Z, Li Q, Bu H, Lin F (2010). Mesenchymal stem cells inhibit complement activation by secreting factor H. Stem Cells Dev.

[58] Manthey HD, Woodruff TM, Taylor SM, Monk PN (2009). Complement component 5a (C5a). Int J Biochem Cell Biol.

[59] Lappas M, Woodruff TM, Taylor SM, Permezel M (2012). Complement C5A regulates prolabor mediators in human placenta. Biol Reprod.

[60] Liszewski MK, Atkinson JP (2015). Complement regulator CD46: genetic variants and disease associations. Hum Genomics.

[61] Jeon H, Lee JS, Yoo S, Lee MS (2014). Quantification of complement system activation by measuring C5b-9 cell surface deposition using a cell-ELISA technique. J Immunol Methods.

[62] Crémet L, Broquet A, Brulin B, Jacqueline C, Dauvergne S, Brion R, Asehnoune K, Corvec S, Heymann D, Caroff N (2015). Pathogenic potential of Escherichia coli clinical strains from orthopedic implant infections towards human osteoblastic cells. Pathog Dis.

[63] Connell SR, Tracz DM, Nierhaus KH, Taylor DE (2003). Ribosomal protection proteins and their mechanism of tetracycline resistance. Antimicrob Agents Chemother.

[64] Hammes WP, Neuhaus FC (1974). On the mechanism of action of vancomycin: inhibition of peptidoglycan synthesis in Gaffkya homari. Antimicrob Agents Chemother.

[65] Skripkin E, McConnell TS, DeVito J, Lawrence L, Ippolito JA, Duffy EM, Sutcliffe J, Franceschi F (2008). R chi-01, a new family of oxazolidinones that overcome ribosome-based linezolid resistance. Antimicrob Agents Chemother.

[66] Kielian T, Esen N, Liu S, Phulwani NK, Syed MM, Phillips N, Nishina K, Cheung AL, Schwartzman JD, Ruhe JJ (2007). Minocycline modulates neuroinflammation independently of its antimicrobial activity in staphylococcus aureus-induced brain abscess. Am J Pathol.

