# Supplementary figures and images for: Minocycline modulates NFκB phosphorylation and enhances antimicrobial activity against Staphylococcus aureus in mesenchymal stromal/stem cells
Source: Stem Cell Res Ther. 2017 Jul 21;8:171. doi: 10.1186/s13287-017-0623-1 (PMC5521110; doi:10.1186/s13287-017-0623-1)

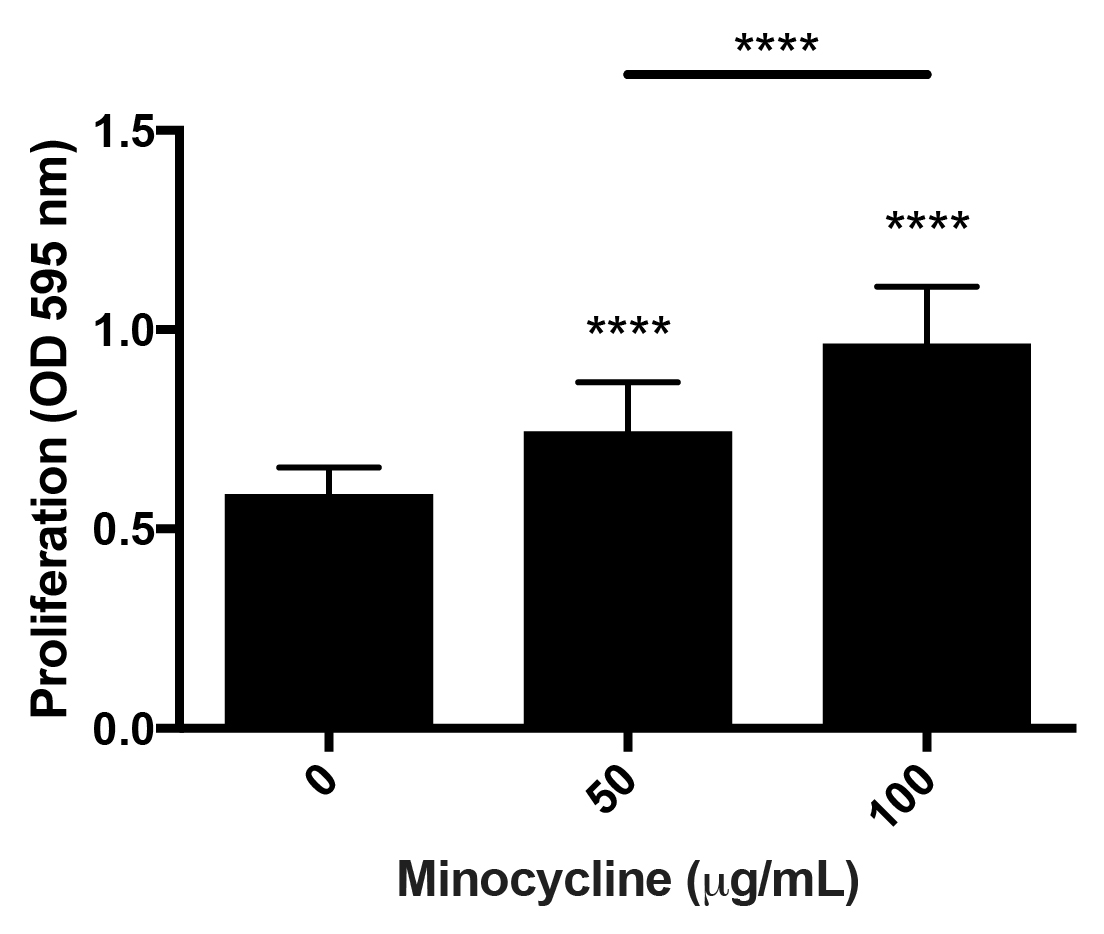

Supplement: Supplementary file 1 — Showing proliferation of MSCs after 24 hours of minocycline treatment in a dose-dependent manner. (JPG 103 kb) [file 13287_2017_623_MOESM1_ESM.jpg]

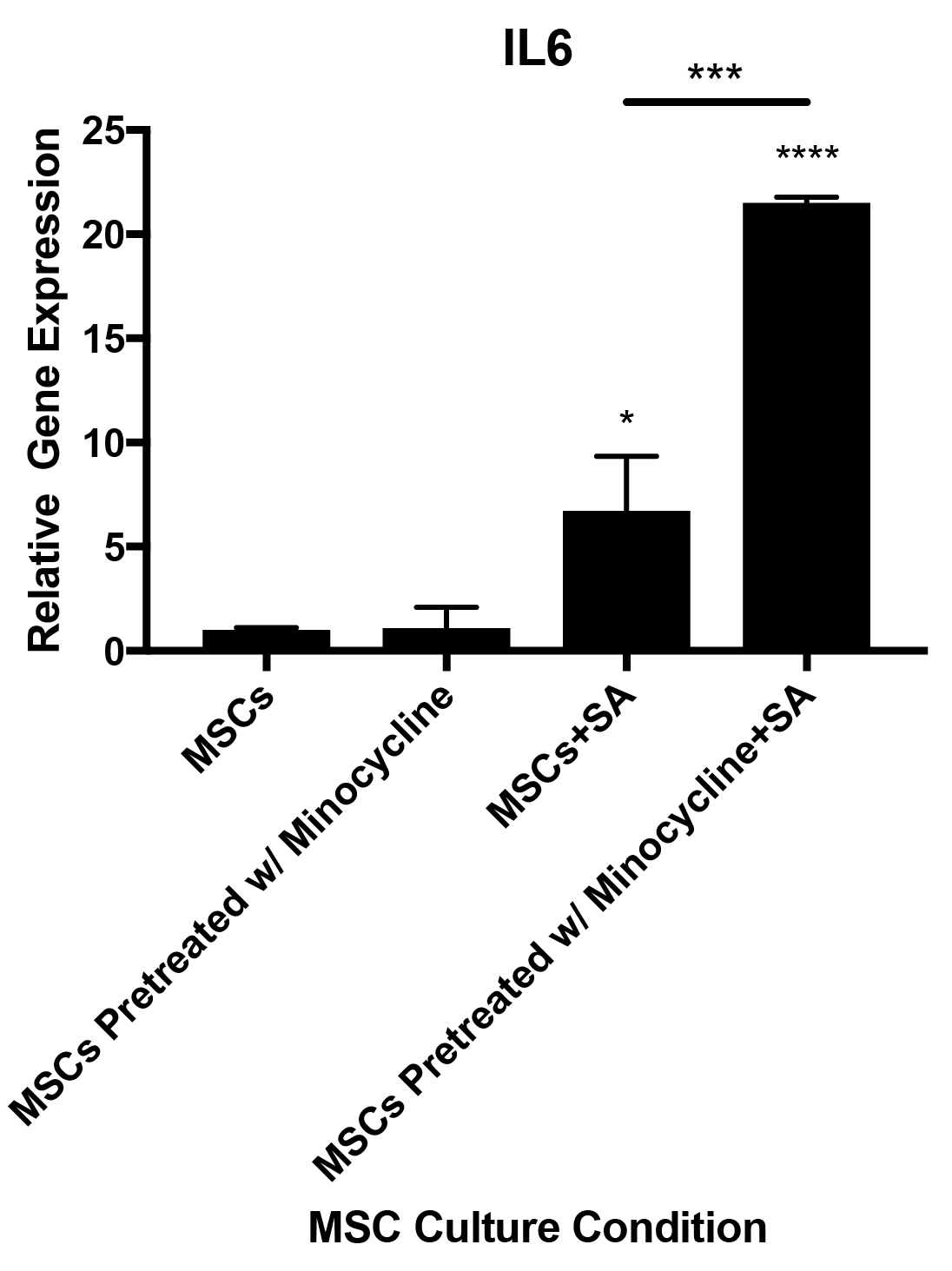

Supplement: Supplementary file 2 — Showing expression of IL6 in MSCs when cultured alone or with SA with and without minocycline pretreatment. (JPG 171 kb) [file 13287_2017_623_MOESM2_ESM.jpg]

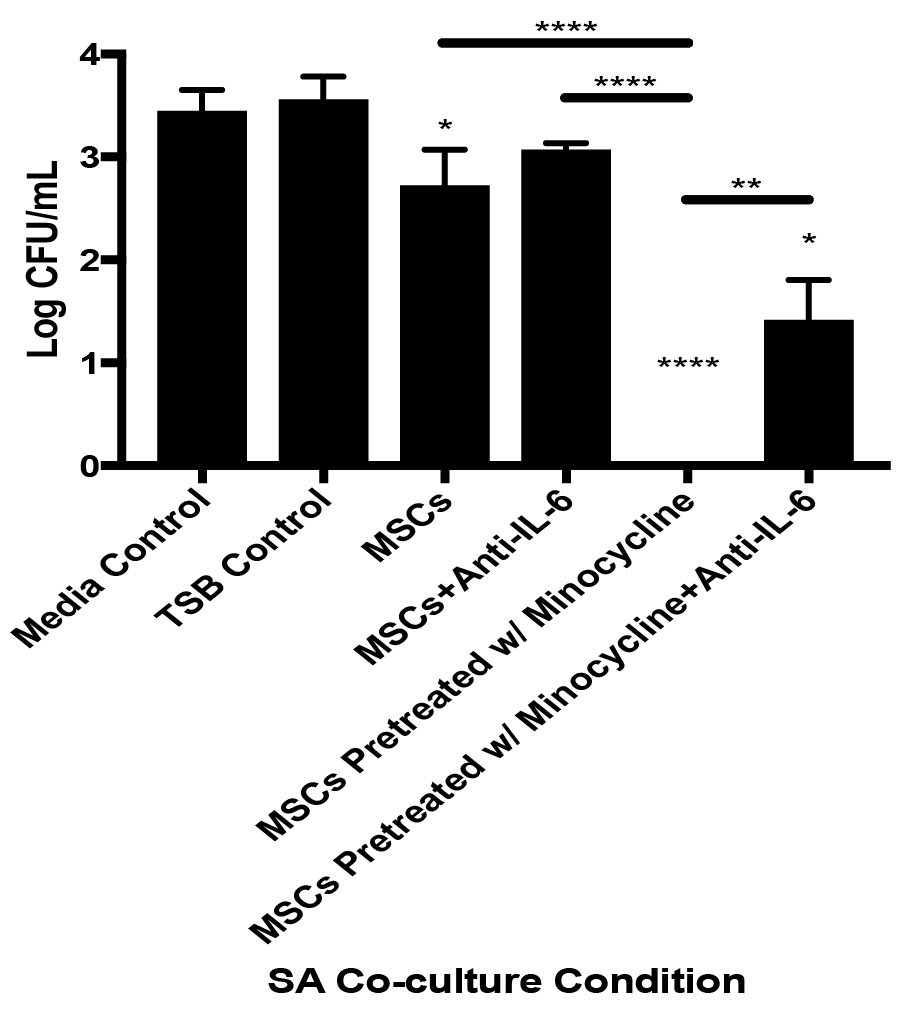

Supplement: Supplementary file 3 — Showing colony forming abilities of SA after 6 hours of coculture with media controls, MSCs with or without anti-IL-6, and MSCs pretreated with minocycline with or without anti-IL-6. (JPG 146 kb) [file 13287_2017_623_MOESM3_ESM.jpg]
